# Household food insecurity is associated with obesogenic health behaviours among a low-income cohort of pregnant women in Boston, MA

**DOI:** 10.1017/S1368980022000714

**Published:** 2023-05

**Authors:** Erika R Cheng, Mandy Luo, Meghan Perkins, Tiffany Blake-Lamb, Milton Kotelchuck, Alexy Arauz Boudreau, Elsie M Taveras

**Affiliations:** 1 Division of Children’s Health Services Research, Department of Pediatrics, Indiana University School of Medicine, 410 W. 10th Street, Suite 2000, Indianapolis, IN 46220, USA; 2 Division of General Academic Pediatrics, Department of Pediatrics, Massachusetts General Hospital for Children, Boston, MA, USA; 3 Department of Obstetrics and Gynecology, Massachusetts General Hospital, Boston, MA, USA; 4 Kraft Center for Community Health, Massachusetts General Hospital, Boston, MA, USA; 5 Department of Nutrition, Harvard T.H. Chan School of Public Health, Boston, MA, USA

**Keywords:** Obesity, Pregnancy, Nutrition, Household food insecurity, Health behaviours, Boston, MA

## Abstract

**Objective:**

To examine associations of household food insecurity with health and obesogenic behaviours among pregnant women enrolled in an obesity prevention programme in the greater Boston area.

**Design:**

Cross-sectional evaluation. Data were collected from structured questionnaires that included a validated two-item screener to assess household food insecurity. We used separate multivariable linear and logistic regression models to quantify the association between household food insecurity and maternal health behaviours (daily consumption of fruits and vegetables, sugar-sweetened beverages and fast food, physical activity, screen time, and sleep), mental health outcomes (depression and stress), hyperglycaemia status and gestational weight gain.

**Setting:**

Three community health centres that primarily serve low-income and racial/ethnic minority patients in Revere, Chelsea and Dorchester, Massachusetts.

**Participants:**

Totally, 858 pregnant women participating in the First 1,000 Days program, a quasi-experimental trial.

**Results:**

Approximately 21 % of women reported household food insecurity. In adjusted analysis, household food insecurity was associated with low fruit and vegetable intake (*β* = −0·31 daily servings; 95 % CI −0·52, −0·10), more screen time (*β* = 0·32 daily hours; 95 % CI 0·04, 0·61), less sleep (*β* = −0·32 daily hours; 95 % CI −0·63, −0·01), and greater odds of current (adjusted odds ratio (AOR) 4·42; 95 % CI 2·33, 8·35) or past depression (AOR 3·01; 95 % CI 2·08, 4·35), and high stress (AOR 2·91; 95 % CI 1·98, 4·28).

**Conclusions:**

In our sample of mostly low-income, racial/ethnic minority pregnant women, household food insecurity was associated with mental health and behaviours known to increase the likelihood of obesity.

The global obesity prevalence continues to rise among children and adults^(1,2)^, emphasising the need to identify mutable risk factors for targeted intervention. A compelling and growing body of evidence indicates that the roots of obesity and many related adult diseases have their origins in early childhood, or even before^(3)^. Pregnancy thus offers ample opportunity for prevention efforts, as women’s dietary habits, gestational weight gain and prenatal health behaviours have all consistently and significantly been associated with women’s future weight status^(4)^, as well as with offspring weight^(5)^.

As such, the American College of Obstetrics and Gynecologists recommends that women maintain healthy diets and engage in physical activity to avoid gaining excessive weight while pregnant^(6)^. Unfortunately, half of US women have overweight or obesity when they enter pregnancy and 47 % gain more weight than the recommended limits^(7)^; pre-pregnancy BMI and gestational weight gain in turn have been linked with both negative impacts for women’s future health and the likelihood for obesity in their children^(3)^. Moreover, racial/ethnic minority and low-income women are disproportionately more likely to enter pregnancy with unhealthy weights and gain more weight during pregnancy than their counterparts^(8)^, further exacerbating existing health inequities for their children.

Low-income women are also more likely to live in households experiencing food insecurity, defined as having limited or uncertain availability of nutritionally adequate and safe foods or the ability to acquire such foods in socially acceptable ways^(9)^. Paradoxically, food insecurity often coexists with obesity, especially among women^(10)^. During the pregnancy period, food insecurity is linked with greater maternal pre-pregnancy BMI^(11)^, higher maternal gestational weight gain^(12)^, gestational diabetes^(13)^ and low birth weight^(13)^. Although the exact mechanisms are likely multifaceted, diet-related behaviours may represent a potential pathway linking household food insecurity with these outcomes; however, few published studies have examined associations of food insecurity with adverse diet and health-related behaviours in pregnancy that contribute to the development of obesity and its consequences in mother–infant dyads and their families.

Increasing our knowledge of the obesity-related health implications of food insecurity during pregnancy could inform clinical and public health efforts to maximise individual and population-based interventions. Such work is especially timely given recent data demonstrating a dramatic increase in the percent of families reporting food insecurity during the COVID-19 pandemic^(14)^. Therefore, our goal was to examine the association of household food insecurity with obesogenic behaviours, mental health and health factors in a sample of low-income, pregnant women enrolled in an obesity prevention programme.

## Methods

### Study design and population

Our data were derived from a cross-sectional survey of women participating in the First 1,000 Days program, a quasi-experimental trial situated within three community health centres in Massachusetts. The conceptual framework, intervention design and evaluation methods of the First 1,000 Days program have been described in detail elsewhere^(15)^. Briefly, the First 1,000 Days program is a systems-level initiative aimed to reduce the prevalence of overweight by addressing individual, family and socio-contextual factors across early life clinical and public health systems. Between 9 August 2016 and 29 September 2017, the programme recruited 885 pregnant women receiving care at three community health centres that primarily serve low-income and racial/ethnic minority patients in Revere, Chelsea and Dorchester, Massachusetts.

**Fig. 1:**
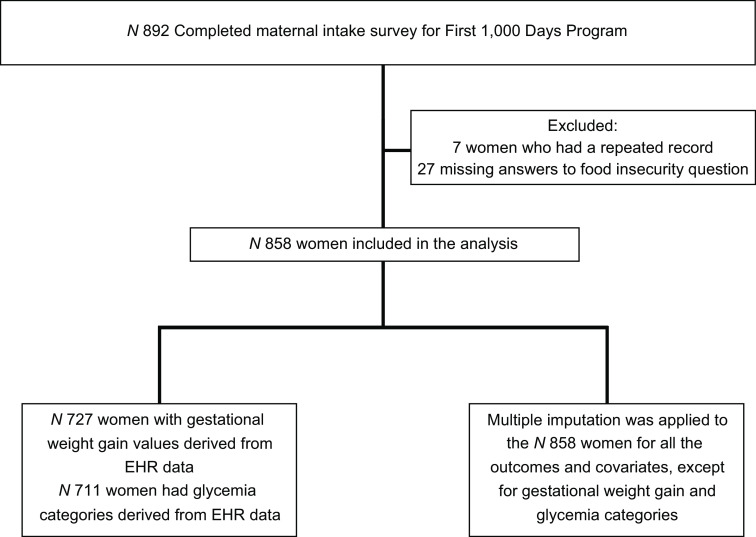
Sample Size Flow

Our sample consists of 858 women who enrolled in the programme and who completed a baseline questionnaire during intake (approximately 11 weeks’ gestation; mean 10·8 weeks, range 3–39 weeks) and answered the food insecurity screening questions. We excluded twenty-seven women from analyses who did not respond to the food insecurity questions (see Fig. 1).

### Measures

#### Household food insecurity status

Household food insecurity was assessed using the Hunger Vital Sign^TM^, a validated two-item screening tool^(16)^ that queries how often the household ‘worried whether our food would run out before we got money to buy more’; and ‘how often the food we bought just didn’t last and we didn’t have money to get more’. Participants responded on a three-point Likert scale, including ‘often true’, ‘sometimes true’ and ‘never true’. We defined households as food-insecure if participants responded ‘often true’ or ‘sometimes true’ to either question.

#### Obesogenic behaviours

Women responded to one question regarding frequency of each of the following behaviours: fruit and vegetable, sugar-sweetened beverages (SSB) and fast-food consumption. The item about fruit/vegetable consumption asked: ‘During the past 7 d, on average, how often did you eat fruit or vegetables (including fresh, cooked, canned, or frozen)? Do not include fruit or vegetable juice or dried fruits’. The item about SSB consumption asked: ‘During the past 7 d, on average, how often did you drink 100 % fruit juice or a sugar-sweetened beverage? Sugar-sweetened beverages are things like fruit-flavoured drinks, juice from concentrate, punch, Kool-Aid, soda, sports drinks, sweet tea or coffee drinks, or sweetened milks’. The item about fast-food consumption asked: ‘During the past 7 d, on average, how often did you eat something from a fast food restaurant? Examples include McDonald’s, Burger King, Taco Bell, Subway’. For all food and beverage consumption items, response options included 0 = never, 1 = once/week, 2 = 2–4 times/week, 3 = nearly daily or daily, 4 = 2–4 times/d or 5 = 5 or more times a day. We converted responses into servings per d.

We evaluated physical activity by asking mothers the question: ‘In the past 7 d, how many days were you physically active for a total of at least 30 min/d?’ Screen time was assessed by the question: ‘During the past 7 d, on average, how many hours per day did you usually spend watching TV or videos? Include time spent watching on a television, computer, phone or tablet’. Sleep duration was evaluated with the question: ‘During the past 7 d, on average, how many hours of sleep did you get in a 24-h period?’

#### Mental health

We assessed past depression using two questions: (1) ‘Before this pregnancy, was there ever a period of time when you were feeling depressed or down or when you lost interest in pleasurable activities most of the day, nearly every day, or for at least 2 weeks?’^(17)^ and (2) ‘Before this pregnancy, did you ever see a health care professional who said that you were depressed?’^(18)^ We coded women has having past depression if they answered yes to either of these questions.

To measure current depression, we extracted electronic medical record (EMR) data from the Patient Health Questionnaire-2 (PHQ-2) and the Edinburgh Postnatal Depression Scale (EPDS). The PHQ-2 is a validated two-question depression tool that measures the frequency of depressed mood and anhedonia over the past 2 weeks^(19)^. The EPDS is a 10-question screening tool that was developed to identify women with prenatal depression^(20)^. We flagged mothers with PHQ-2 scores ≥ 3 or EPDS scores ≥ 13 as having current depression.

Maternal stress was assessed by a single question adapted from the Growing Up Today Study^(21)^: ‘How much stress do you feel in your life’? We coded mothers who responded ‘I feel stress fairly often’, ‘I sometimes feel a lot of stress’ or ‘I feel a lot of stress most of the time’ as having high stress.

Pregnancy anxiety was assessed using a five-item tool developed by Rini *et al*.^(22)^ which asked mothers to report how often they felt concerned or worried about the following (1 = never, 4 = almost all of the time): (1) How the baby is growing or developing inside of me; (2) Losing the baby; (3) Having a hard or difficult labour and delivery; (4) Taking care of the new baby and (5) Developing medical problems during my pregnancy. We calculated the total pregnancy anxiety score by adding score of each question, with higher scores indicating more pregnancy-related anxiety.

#### Health factors

Maternal hyperglycaemia status and gestational weight gain were calculated using EMR data. Following the approach of Huang *et al*.^(23)^, we defined categories of pregnancy hyperglycaemia status using results from both women’s 1-h non-fasting glucose challenge test (GCT) and their 3-h fasting oral glucose tolerance test (OGTT). We flagged women as having normal glucose tolerance if their GCT level was <=140 mg/dl. For women with GCT levels >140 mg/dl, we extracted additional OGTT data, defining abnormal OGTT as >95 mg/dl at baseline, >180 mg/dl at 1 h, >155 mg/dl at 2 h and >140 mg/dl at 3 h, following American Diabetes Association (ADA) criteria^(24)^. We then defined women’s hyperglycaemia status as follows: (1) normal glucose tolerance when GCT glucose level ≤140 mg/dl; (2) isolated hyperglycaemia when a GCT level >140 mg/dl was followed by normal OGTT for all four measures as described above; (3) impaired glucose tolerance when a GCT glucose level >140 mg/dl was followed by an OGTT with one abnormal result as described above and (4) gestational diabetes mellitus when a GCT blood glucose level >140 mg/dl was followed by an OGTT with ≥2 abnormal results as described above.

Finally, gestational weight gain was calculated by subtracting maternal weight measured or self-reported in the first trimester from the last maternal weight collected before delivery. If maternal weight in first trimester was not available, we used mother’s weight within 9 months prior to the pregnancy^(15)^.

### Covariates

We collected data on key maternal demographic characteristics via the baseline questionnaire, including self-reported race/ethnicity (White, Black/African American, Asian/other and Hispanic/Latino), marital status (unmarried/living together *v*. unmarried), employment (full time, part time and unemployed), annual household income (<$10 000, $10 001–$20 000, $20 001–$50 000 and >$50 000) and country of birth (USA *v*. other). Maternal education (some high school or less, high school graduate and more than high school/other) was extracted from the EMR. We augmented missing data from the baseline questionnaire using data from the EMR.

### Statistical analysis

Analyses were conducted using SAS v9.4. We summarised participant characteristics using descriptive statistics and examined factors associated with household food insecurity using bivariate analyses. We then quantified the unadjusted association of household food insecurity and each factor using linear regression models for continuous factors (fruit and vegetable intake, SSB intake, fast-food intake, physical activity, screen time, sleep, total anxiety score, gestational weight gain and GCT value) and logistic regression models for dichotomous factors (current/past depression, high stress, GWG categories and glycemia categories; Model 1). Separate multivariable linear and logistic regression models then quantified the same associations adjusted for maternal race/ethnicity, annual household income, marital status and maternal education. We adjusted for race/ethnicity given its association with both food insecurity and obesity^(25)^. Because some variables were missing up to 20 % of data, we performed multiple imputation analysis with a fully conditional specification option in SAS. Regression analyses were performed on ten imputed datasets, which we combined using PROC MIANALYZE. We did not impute missing gestational weight gain and gestational diabetes because we were unable to confirm if women were missing data due to delivery outside the MassGeneral Brigham system, or if the pregnancy was lost or terminated before delivery.

## Results

Table 1 presents demographic characteristics of the overall sample, and comparing those who reported and who did not report household food insecurity. Our sample of 858 pregnant women was predominantly Hispanic or Latina (50·1 %) with a mean age of 29·1 years (sd 5·8 years) at the time of the baseline survey. Most women were married or reported living with a partner (80·5 %), had a high school education or greater (78·0 %) and were born outside of the USA (63·9 %). Approximately one-third (34·5 %) were unemployed and 40·1 % reported annual household incomes of less than $20 000.

**Table 1:** Demographic characteristics, obesogenic behaviours, mental health and health factors of the sample, overall and by household food insecurity status (*n* 858)

			Reported household food insecurity				*P*-value
	Full sample overall		No		Yes		
	*n* 858		*n* 677		*n* 181		
	*n*	%	*n*	%	*n*	%	
Demographic characteristics							
Race/ethnicity (*n* 857)							
White	241	28·1	213	31·5	28	15·5	<0·001
Black or African American	88	10·3	64	9·5	24	13·3	
Asian or other	99	11·6	80	11·8	19	10·5	
Hispanic or Latino	429	50·1	319	47·2	110	60·8	
Marital status (*n* 856)							
Married/living together	689	80·5	561	83·0	128	71·1	<0·001
Unmarried	167	19·5	115	17·0	52	28·9	
Employment (*n* 854)							
Employed full-time	321	37·6	279	41·4	42	23·3	<0·001
Employed part-time	238	27·9	185	27·4	53	29·4	
Unemployed	295	34·5	210	31·2	85	47·2	
Annual household income (*n* 775)							
Less than $10 000 yearly	127	16·4	79	12·7	48	31·2	<0·001
$10 001 to $20 000 yearly	184	23·7	137	22·1	47	30·5	
$20 001 to $50 000 yearly	300	38·7	248	39·9	52	33·8	
Greater than $50 000 yearly	164	21·2	157	25·3	7	4·5	
Education (*n* 685)							
Some high school or less	151	22·0	102	18·8	49	34·8	<0·001
High school graduate	256	37·4	196	36·0	60	42·6	
More than high school or other	278	40·6	246	45·2	32	22·7	
Birth country							
USA	310	36·1	254	37·5	56	30·9	0·10
Other	548	63·9	423	62·5	125	69·1	
Intervention site							
Chelsea	395	46·0	295	43·6	100	55·2	<0·001
Revere	378	44·1	323	47·7	55	30·4	
Dorchester	85	9·9	59	8·7	26	14·4	

	Mean	sd	Mean	sd	Mean	sd	
Household size (*n* 807)	3·9	1·6	3·8	1·6	4·0	1·5	0·21
Age at enrolment, years (*n* 750)	29·1	5·8	29·4	5·6	27·9	6·6	0·01
Age at delivery, years (*n* 743)	29·6	5·8	30·0	5·6	28·3	6·6	0·005
Obesogenic behaviours							
Fruit/veg intake (servings/d)	1·38	1·3	1·47	1·3	1·04	1·1	<0·001
SSB intake (servings/d)	0·75	0·95	0·75	0·97	0·78	0·89	0·67
Fast-food intake (servings/d)	0·17	0·36	0·16	0·34	0·20	0·42	0·26
Physical activity (d/week)	2·74	2·4	2·74	2·4	2·76	2·8	0·92
Screen time (h/d)	2·36	1·7	2·28	1·6	2·64	1·9	0·02
Sleep (h/d)	7·51	1·8	7·57	1·7	7·28	2·1	0·08
	*n*	%	*n*	%	*n*	%	
Mental health							
Current depression (*n* 720)	42	5·8	21	3·7	21	13·9	<0·001
Past depression (*n* 858)	246	28·7	157	23·2	89	49·2	<0·001
High stress (*n* 856)	229	26·8	154	22·8	75	41·4	<0·001

	Mean	sd	Mean	sd	Mean	sd	
Total anxiety score	9·22	3·0	8·93	2·8	10·34	3·5	<0·001
Health factors							
Gestational weight gain (kg) (*n* 727)	11·61	6·3	11·74	6·2	11·12	6·7	0·30
GCT value (mg/dl) (*n* 707)	118·4	29·0	118·57	28·7	117·66	30·3	0·74
	*n*	%	*n*	%	*n*	%	
Hyperglycaemia status (*n* 710)							
Normal glucose tolerance	577	81·3	460	81·4	117	80·7	0·47
Isolated hyperglycaemia	63	8·9	53	9·4	10	6·9	
Impaired glucose tolerance	24	3·4	19	3·4	5	3·4	
GDM	46	6·5	33	5·8	13	9·0	
Gestational diabetes (*n* 711)	46	6·5	33	5·8	13	9·0	0·17
Gestational weight gain (*n* 727)							
Excessive	269	37·0	211	36·6	58	38·4	0·51
Normal	269	37·0	219	38·0	50	33·1	
Inadequate	189	26·0	146	25·3	43	28·5	

SSB, sugar sweetened beverages; GCT, glucose challenge test; GDM, gestational diabetes mellitus.

Over 21 % of women (*n* 181) resided in food-insecure households. Relative to women who did not report household food insecurity, these women were younger (mean age 27·9 *v*. 29·4 years), and more likely to be Hispanic (60·8 % *v*. 47·2 %), unmarried (28·9 % *v*. 17·0 %), less educated (34·8 % *v*. 18·8 % reporting less than high school education), unemployed (47·2 % *v*. 31·2 %) and report lower annual household incomes (31·2 % *v*. 12·7 % reporting annual incomes of less than $10 000).

Table 1 also presents the prevalence of maternal obesogenic behaviours, mental health and health factors for the entire sample, and by household food insecurity status. In the full overall sample, women reported consuming approximately 1·38 servings of fruit and vegetables, 0·75 servings of SSB and 0·17 fast-food servings per d. They reported over 2·36 h of daily screen time and an average of 7·51 h of daily sleep. Nearly one in three (28·7 %) reported having a history of depression with 5·8 % screening positive for depression at the time of the survey. Total anxiety scores averaged 9·2 (range 4–20). Compared with women who did not report household food insecurity, women living in food-insecure households consumed fewer fruits and vegetables per d (1·0 *v*. 1·5 servings, *P*-value <0·001) and reported more hours of daily screen time (2·6 h *v*. 2·3 h, *P*-value <0·02) and higher levels of anxiety (10·3 *v*. 8·9, *P*-value <0·001). Women from food-insecure households were also more likely to have depression, both past (49·2 % *v*. 23·2 %, *P*-value <0·001) and current (13·9 % *v*. 3·7 %, *P*-value <0·001), as well as report high stress (41·4 % *v*. 22·8 %, *P*-value <0·001) than women from households without food insecurity. There were no statistically significant group differences in SSB intake, fast-food intake, physical activity, sleep, gestational weight gain or glycemia categories.

Table 2 shows the the unadjusted bivariate and multivariate-adjusted associations of household food insecurity with these factors of interest. Mean differences and associated 95 % CI are presented for continuous outcomes and ORs are presented for dichotomous outcomes; βs and ORs represent the mean differences in or odds of each outcome, respectively, between women who reported household food insecurity and those who did not. Multivariate-adjusted models control for maternal race/ethnicity, annual household income, marital status and maternal education. As displayed in the table, after adjustment, household food insecurity was significantly associated with lower fruit and vegetable intake (*β* = −0·31 daily servings; 95 % CI −0·52, −0·10), more daily screen time (*β* = 0·32 h; 95 % CI 0·04, 0·61) and less daily sleep (*β* = −0·32 h; 95 % CI −0·63, −0·01). In terms of our dichotomous outcomes, household food insecurity was associated with increased odds of reporting current depression (adjusted odds ratio (AOR) 4·42; 95 % CI 2·33, 8·35), past depression (AOR 3·01; 95 % CI 2·08, 4·35) and high stress (AOR 2·91; 95 % CI 1·98, 4·28).

**Table 2:** Unadjusted and adjusted associations of household food insecurity with obesogenic behaviours, mental health and health factors during pregnancy, *n* 858

	Unadjusted (bivariate)		Multivariate-adjusted*	
	Mean difference	95 % CI	Mean difference	95 % CI
Continuous outcomes				
Fruit and veg intake (servings/d)	**−0·43**	−**0·64,** −**0·23**	**−0·31**	−**0·52,** −**0·10**
SSB intake (servings/d)	0·03	−0·13, 0·19	−0·06	−0·22, 0·11
Fast-food intake (servings/d)	0·04	−0·02, 0·10	0·01	−0·05, 0·07
Physical activity (d/week)	0·01	−0·38, 0·40	0·10	−0·31, 0·51
Screen time (h/d)	**0·36**	**0·09, 0·63**	**0·32**	**0·04, 0·61**
Sleep (h/d)	**−0·30**	−**0·60,** −**0·01**	**−0·32**	−**0·63,** −**0·01**
Total anxiety score	**1·43**	**0·94, 1·92**	**1·55**	**1·04, 2·05**
Gestational weight gain (kg) (*n* 727)	−0·62	−1·75, 0·50	−0·56	−1·73, 0·61
GCT value (mg/dl) (*n* 707)	−0·91	−6·20, 4·38	0·01	−5·46, 5·48
Dichotomous outcomes	OR	95 % CI	OR	95 % CI
Current depression (reference = no)	**4·21**	**2·30, 7·69**	**4·42**	**2·33, 8·35**
Past depression (reference = no)	**3·20**	**2·28, 4·51**	**3·01**	**2·08, 4·35**
High stress (reference = no)	**2·40**	**1·70, 3·39**	**2·91**	**1·98, 4·28**
GWG categories				
Normal	1·00	Reference	1·00	Reference
Inadequate	1·29	0·82, 2·04	1·17	0·72, 1·90
Excessive	1·20	0·79, 1·84	1·14	0·73, 1·79
Glycemia categories				
Normal glucose tolerance	1·00	Reference	1·00	Reference
Isolated hyperglycaemia	0·74	0·37, 1·50	0·89	0·42, 1·89
Impaired glucose tolerance	1·03	0·38, 2·83	1·04	0·36, 3·02
GDM	1·55	0·79, 3·04	1·60	0·79, 3·26

SSB, sugar sweetened beverages; GCT, glucose challenge test; GWG, gestational weight gain; GDM, gestational diabetes mellitus.

* Adjusted models control for maternal race/ethnicity, annual household income, marital status and education.

Multiple imputation was applied to impute the missingness in outcomes, predictor and covariates, except for GWG and GDM, and related categorical outcomes.

Values in bold are statistically significant at *P* < 0·05.

Reference group for the regression models is women who did not report household food insecurity.

Logistic regression was applied for dichotomous/categorical outcomes and linear regression was applied to continuous outcomes.

β-coefficients and OR represent the mean difference in or odds of each factor for women reporting household food insecurity compared with women who did not report household food insecurity.

## Discussion

Approximately 21 % of the mostly low-income, racial/ethnic minority pregnant women in our sample reported household food insecurity, which is greater than the 10·5 % of overall US households in 2019^(26)^. Relative to women from households without food insecurity, women with food insecurity in our sample were younger, and more likely to be Hispanic, unmarried, less educated, and unemployed, and report lower annual household incomes. In turn, pregnant women living in food-insecure households were significantly more likely to report poor dietary behaviours including low fruit and vegetable intake even after adjusting for multiple potential confounding factors. This is consistent with other studies^(27,28)^. Our multivariable model also showed that pregnant women with household food insecurity were more likely to report obesogenic-promoting behaviours including less daily sleep, more daily screen time, current or past depression, and high stress.

Our results parallel prior cross-sectional research on household food insecurity and dietary intake in the adult population and lend new insight into how household food insecurity affects development across the lifespan, especially among pregnant women. Consistent with findings from non-pregnant populations, household food insecurity disproportionately affected pregnant women in our sample with the highest risk for obesity, including low-income women and members of racial or ethnic minority groups^(29)^. These women were also more likely to engage in obesity-promoting health behaviours that track across the life course, thereby highlighting potentially important mechanisms linking food insecurity during pregnancy to childhood obesity, and racial/ethnic and socio-economic disparities therein. In related research, household food insecurity during pregnancy is associated with obesity-promoting maternal attitudes, such as lower locus of control over the prevention of childhood obesity^(30)^ and decreased self-efficacy to make fruit and vegetables available for children^(31)^. Here, we show that household food insecurity is also associated with specific prenatal health behaviours that contribute to postpartum weight retention^(32)^ and offspring weight^(5)^, like low fruit and vegetable intake. Other work shows the impact of household food insecurity on obesity-promoting feeding styles and practices, including pressuring, indulgent and laissez-faire feeding styles^(27)^, supplementing breastmilk with formula, limiting healthy foods like fruits, vegetables and lean meats, and increasing juice^(33)^. Our study highlights the role of household food insecurity in promoting obesogenic behaviours for women and family earlier in life course, thereby highlighting a critical time period for intervention.

Pregnant women with household food insecurity in our sample were also over four times more likely to report depression and nearly three times more likely to report high stress than those not reporting household food insecurity, after adjusting for demographic and socio-economic characteristics. These results parallel previous cross sectional research^(34)^. Food-insecure women also reported fewer hours of daily sleep and increased screen time, which are both linked with increased obesity risk^(35)^. While the mechanisms are unclear, household food insecurity during pregnancy can be conceptualised as a poverty-related stressor that leads to the development of mental health problems and increases the risk for suboptimal sleep. The link between maternal depression and poor nutritional intake during childhood^(36)^ as well as with childhood obesity^(37)^ indicates that interventions aimed at improving household food security for pregnant women are likely to have positive effects on the mental and physical well-being of both mothers and children.

Strengths of our study include the use of an existing cohort of pregnant women participating in a large quasi-experimental trial, which allowed us to adjust for other known risk factors and to use validated questionnaires to assess household food insecurity status, maternal obesogenic-promoting behaviours, mental health and health factors. However, we also acknowledge limitations including the use of cross-sectional data, which did not allow us to untangle the directionality of the relationships we describe. For example, it is possible that maternal mental health (e.g. depression) mediated of the relationship between household food insecurity and some of our obesogenic behaviours like increased screen time. Others suggest that the relationship among maternal depression and household food insecurity over time is bidirectional^(36)^. In other words, depression or stress in women could lead to socio-economic difficulties such as reduced education or employment, ultimately leading to food insecurity. Future research is needed to model these relationships using longitudinal data. Our sample of mostly low-income racial/ethnic minority women also limits generalisability to all pregnant women.

Understanding how household food insecurity affects development across the lifespan is critical to informing clinical and public health preventive efforts and is especially timely given dramatic increases in the number of US households reporting food insecurity during the COVID-19 pandemic^(14)^. Our results suggest that obesity prevention strategies should recognise household food insecurity as an important early antecedent and consider its effect on obesity-promoting behaviours as early in the life course as possible. Pregnancy, in particular, could be viewed as a time in which food insecurity is worthy of assessment and target for programming. Interestingly, although associations of household food insecurity with behaviours known to be associated with obesity were found in our sample, we did not note a direct association of food insecurity with gestational weight gain. Instead, our findings point to the potential role of stress, depression, screen time and poor diet quality as major factors related to household food insecurity during this time frame that could negatively impact obesity outcomes for women and children. Therefore, a two-pronged approach that targets nutrition and activity-related obesogenic behaviours as well as high stress and depression during pregnancy for food-insecure women may be a promising approach to stem the rising rates of infant and maternal obesity and overweight. Given substantial evidence that household food insecurity and obesity coexist, it is critical that efforts to ameliorate household food insecurity do not unintentionally promote excess weight gain but instead consider opportunities to promote healthy behaviour (e.g. fruit and vegetable intake).

In conclusion, this study highlights the associations of household food insecurity during pregnancy with behaviours known to increase the likelihood of obesity within families. There is an urgent need to identify policies that simultaneously address household food insecurity and obesity as the prevalence of obesity continues to rise among US families.
